# Cultural fiction as predictive-affective simulation: a neurocognitive model of male emotional regulation and gender violence prevention

**DOI:** 10.3389/fpsyg.2026.1757506

**Published:** 2026-04-22

**Authors:** Maria Tartari, Alice Conti, Giulia Candeloro, Lorenza Lucchi Basili, Sara Uboldi, Pier Luigi Sacco

**Affiliations:** 1 Istituto di Scienze del Patrimonio Culturale, Consiglio Nazionale delle Ricerche, Naples, Italy; 2 Department of Neurosciences, Imaging and Clinical Science, Università Degli Studi Chieti-Pescara “Gabriele d’Annunzio, Chieti, Italy; 3 Gender Strategies Center, Chieti, Italy; 4 metaLAB (at) Harvard, Cambridge, MA, United States

**Keywords:** cultural fiction, emotion regulation, empathy, feminicide, feminist cultural policy, gendered violence prevention, neuroaesthetics, predictive mind

## Abstract

This conceptual article develops an integrative framework connecting the Tie-Up Theory of affective receptivity with predictive processing models of brain function, in order to advance a neurocognitive understanding of the emotional mechanisms underlying gender-based violence and its prevention. Departing from the observation that feminicide cannot be explained solely through sociological or ideological variables, the paper focuses on its emotional and predictive precursors, particularly the inability to tolerate frustration, rejection, or loss without attempting to restore control. Drawing on research in psychology, cognitive neuroscience, cultural studies, and gender theory, we argue that cultural fictionality (as encountered in theatre, cinema, and literature) can function as a predictive-affective simulator. In this model, Predictive Processing provides the mechanistic account of how emotional states arise through prediction errors and their regulation, while the Tie-Up Theory specifies the affective content that these mechanisms operate upon, describing how the male Receptive Area responds to narrative interiority, vulnerability, and relational complexity. A key contribution of this paper is the identification of a specific intervention window within the documented escalation phases that precede feminicide: the transition between emotional dysregulation (rumination, anger, shame) and maladaptive coping (externalization, aggression). We argue that engagement with cultural fiction may modulate this transition by strengthening emotional flexibility and tolerability of negative affect before it crystallizes into violent scripts. Through symbolic exposure to disappointment, ambiguity, and emotional dissonance, cultural fictions allow individuals to rehearse adaptive regulation within safe aesthetic frames, counteracting the rigid control-oriented schemas that sustain patriarchal violence. Although the model is theoretical, it offers a generative basis for future empirical research in experimental psychology, audience studies, and neurocognitive measurement to assess the potential of cultural fiction as an affective regulatory intervention.

## 1 Introduction

Feminicide, namely the intentional killing of women by intimate partners or former partners, constitutes the most lethal expression of gender-based violence and remains a persistent public health crisis across Western contexts (Spencer, 2025; Koureta et al., 2025). Despite that men make up 81% of homicide victims globally, women account for 66% of intimate partner homicide victims (Spencer, 2025). A growing body of empirical evidence indicates that feminicide cannot be explained solely by sociological or ideological variables: at its core lies also a failure of emotional self-regulation. Across multiple studies, perpetrators exhibit impaired capacity to tolerate frustration, rejection, and relational loss: emotional vulnerabilities that, when unregulated, escalate into coercive control and ultimately lethal violence (Moro et al., 2025; Vignola-Lévesque and Léveillée, 2022; Dokkedahl et al., 2022; Cunha et al., 2024; Grigorian et al., 2020). Crucially, this dysregulation is not simply individual psychopathology: it is systematically produced and reinforced by patriarchal socialization patterns that restrict male emotional expression, penalize vulnerability, and anchor male identity to relational control (Gasparrini, 2016, 2020; Ciccone, 2009). The inability to process negative affect adaptively, particularly in the face of separation, rejection, or perceived abandonment, thus represents both a neuropsychological impairment and a gendered affective conditioning (Gross, 2015).

If emotional dysregulation is a proximate mechanism of feminicide, the question of how it can be addressed at a structural, preventive level becomes urgent. We argue that cultural fictionality, namely the domain of aesthetic and narrative experience produced through the arts, literature, theatre, and cinema, constitutes a privileged medium for the exercise and recalibration of emotional regulation. This claim rests on a convergent body of evidence showing that engagement with fiction activates affective and predictive processes that are functionally equivalent to those operative in real social situations (Oatley, 2016; Mar and Oatley, 2008; Kaufman and Libby, 2012), and that such engagement produces measurable effects on empathy, perspective-taking, and affect tolerance (Koopman and Hakemulder, 2015; Johnson, 2012). Cultural fictionality, in this sense, does not operate as a moral classroom or an instrument of ideological persuasion: it operates as a neurocognitive training ground.

We define cultural fictionality as a device for the exercise of counterfactual thinking: an active mechanism, intrinsic to cultural and artistic practice, that modulates our relationship with reality by generating a cognitive field of complex simulation. Culture, by its very nature, works through fictionality, suspending the principle of reality and activating that of possibility. It is precisely this constitutive fictionality that enables the arts to produce what Oatley (1999) calls “simulations of social worlds”: mental constructions that, while not corresponding to real events, are invested with full cognitive, emotional, and adaptive validity. Within the symbolic safety of the aesthetic frame, cultural fictionality allows individuals to rehearse affective scenarios, encounter emotional dissonance, and practise tolerating prediction errors without the costs of real-world failure.

This capacity is grounded in the co-evolutionary relationship between narrative practices and human affective development (Dissanayake, 2008; Hernadi, 2002; Boyd, 2009). Cultural and artistic practices, from music and theatre to storytelling and visual arts, generate fictional narratives that function as crystallized repertoires of behavioral and emotional wisdom, making certain dimensions of social interaction that would otherwise remain opaque to conscious reflection accessible to collective experience (Gallese and Guerra, 2015). The works of greatest eudaimonic depth, those that stimulate reflection and affective self-regulation rather than evading tragic or morally complex themes (Oliver and Raney, 2011), access deep structures of human nature that transcend their historical context, functioning at once as cultural products and evolutionary tools.

Building on this convergence, the present article advances an integrative neurocognitive framework connecting the Tie-Up Theory of affective receptivity (Lucchi Basili and Sacco, 2016, 2017) with predictive processing models of brain function (Hohwy, 2013; Seth and Friston, 2016; Ridderinkhof, 2017), in order to specify how cultural fictionality can act as a predictive-affective simulator capable of modulating the transition between emotional dysregulation and maladaptive coping that precedes feminicide. The Tie-Up Theory (detailed in Section 2) provides a sexually dimorphic model of long-term couple formation that identifies a specific psycho-emotional vulnerability in male relational architecture: men form their deepest emotional bonds through mostly sub-conscious compatibility mechanisms (the Male Receptive Area), yet patriarchal socialization provides them the fewest regulatory resources precisely in this psycho-emotional dimension. This asymmetry creates the conditions under which relational disruption generates the catastrophic emotional responses documented in the feminicide literature.

It is important to clarify that no direct causal relationship between cultural consumption and feminicide is proposed here. The framework concerns affective and cognitive precursors: how rigid predictive and emotional patterns, particularly the inability to tolerate frustration, rejection, or loss, can escalate into violence when unregulated, and how cultural fictionality may intervene preventively by recalibrating these patterns before they consolidate into violent scripts. The link is thus mediated and developmental, not deterministic.

The article is structured into four parts. First, we review the epidemiological, psychological, and neurocognitive literature on feminicide, including the Tie-Up Theory framework that identifies specific male emotional vulnerabilities. Second, we introduce predictive processing as a mechanistic account of emotional dysregulation. Third, we argue that cultural fictionality can act as a predictive-affective simulator that may enhance emotional flexibility. Finally, we discuss the implications for feminist cultural policy and preventive educational programs.

## 2 Emotional dysregulation models: evidences on perpetrators in literature

Recent empirical literature emphasizes that feminicide cannot be reduced to a single cause but rather emerges from a multifactorial interplay. While it has often been analysed through sociocultural, legal, and demographic frameworks, emerging psychological research underscores the pivotal role of emotional regulation processes in the pathway leading to lethal violence. Individual-level vulnerabilities among perpetrators are a consistent finding across diverse contexts. Studies in Sweden and Spain show that major mental disorders, including psychosis and depression, are strongly associated with feminicide (Lysell et al., 2016). Histories of violent crime similarly increase risk, suggesting that feminicide often arises within pre-existing trajectories of aggression rather than sudden breakdowns (Cechova-Vayleux et al., 2013). High psychopathy scores were rare but linked to shorter relationship durations and rapid escalation to lethal violence (Santos-Hermoso et al., 2022). Antisocial and narcissistic personality features appear more prevalent among “antisocial” perpetrator types, whereas dependent and schizoid traits characterize “normalized” perpetrators who commit violence following emotional breakdowns. Collectively, these findings indicate that mental health issues, violent behavioral histories, and poor affect regulation mechanisms jointly elevate feminicide risk.

At the relational level, intimate partner dynamics are the most robust and consistent predictors of feminicide. Studies across Spain, England, the United States, and Australia confirm that prior intimate partner violence (IPV), whether physical, sexual, or psychological, is one of the strongest antecedents of lethal outcomes (Campbell et al., 2017; Vatnar et al., 2017). The period of separation or threat of separation is particularly critical: estrangement can increase risk severalfold, as perpetrators perceive loss of control as intolerable (Ruiz, 2019). Jealousy, controlling behavior, and stalking further heighten danger, with stalking alone associated with a fourfold increase in feminicide risk (Chopra et al., 2022; Ruiz, 2019). Notably, help-seeking behaviors, such as filing police complaints, were not consistently protective, highlighting systemic limitations in prevention mechanisms (Sanz-Barbero et al., 2016). A substantial body of research identifies socioeconomic disadvantage as a structural amplifier of feminicide risk (Carlsson et al., 2021; Vatnar et al., 2017). Immigrant status is another salient determinant, as migrant women may face cultural isolation, precarious employment, and reduced access to protection services (Sanz-Barbero et al., 2016). Similarly, rural residence increases vulnerability, reflecting limited institutional and social resources (Sanz-Barbero et al., 2016). Beyond individual and social domains, feminicide risk is shaped by cultural norms and institutional arrangements that sustain gender inequality. Cross-national analyses link economic, political, and educational gender gaps to higher feminicide rates (Smithey and Thompson, 2022). Patriarchal norms that normalise male control or condone violence against women remain significant background determinants across all studied regions (Koureta et al., 2025). Firearm access emerged as a particularly strong predictor, with law enforcement membership also associated with increased risk (Sorrentino et al., 2022). Institutional deficiencies, including barriers to help-seeking, weak enforcement of protective laws, and ineffective professional risk assessment, further contribute to lethality (Vatnar et al., 2017).

Moreover, several studies describe feminicide as the outcome of a temporal escalation of emotions rather than a spontaneous outburst. The emotional trajectory of male perpetrators is increasingly understood as a dynamic escalation from fear and loss to anger and aggression, mediated by deficits in emotional regulation and reinforced by hegemonic masculine norms (Di Marco and Sandberg, 2024; Grigorian et al., 2020). Across qualitative, quantitative, and neurobiological studies, emotion dysregulation consistently emerges as a primary psychological mechanism among male perpetrators. In their multi-country qualitative study, Di Marco and Sandberg (2024) observed that feelings of fear, helplessness, and pain evolve into anger when men perceive relational or symbolic threats, such as abandonment, infidelity, or loss of control. Violence, in this sense, becomes a maladaptive attempt to regulate these intolerable affective states and to restore a threatened sense of self-worth. Quantitative evidence supports this interpretation: Di Marco et al. (2024) found significantly higher levels of psychological distress and emotional instability among femicide perpetrators than among other violent offenders, suggesting that poor emotion regulation is a distinguishing characteristic of those who commit gendered killings.

Neuroimaging data further corroborate these findings. Amaoui et al. (2023) identified aberrant connectivity patterns involving the dorsolateral prefrontal cortex in perpetrators of IPV, suggesting reduced capacity to down-regulate emotional arousal through cognitive control. Such neural dysfunctions align with the behavioral observation that perpetrators experience overwhelming emotional states they cannot modulate adaptively, leading to externalized forms of control, including aggression.

Aguilar-Ruiz (2018) and Cáceres (2022) both report sequences beginning with feelings of rejection, abandonment, and jealousy, progressing to anxiety and despair, and culminating in an explosive collapse of cognitive-emotional control. Fahs et al. (2023) interpret such violence as the “emotional transfer of pain,” where perpetrators transform internal suffering into outward aggression as a desperate assertion of agency. The trigger events in these sequences are consistently relational disruptions, situations that activate a perceived existential threat to masculine identity. This escalation underscores the absence of adaptive emotional buffering mechanisms and reflects how personal insecurity, attachment disturbances, and patriarchal entitlement converge in the lead-up to feminicide.

In the absence of effective emotion regulation strategies, perpetrators often resort to maladaptive coping mechanisms. Violence itself frequently functions as a pseudo-regulatory act, momentarily relieving psychological tension while perpetuating a destructive feedback loop (Di Marco and Sandberg, 2024). Substance use, particularly alcohol, further compounds dysregulation, serving both as an avoidance strategy and as a facilitator of aggression (Grigorian et al., 2020). Suicidal ideation or self-harming behavior may also occur as an alternative outlet for emotional collapse (Aguilar-Ruiz, 2018). This dominance of externalizing over internalizing regulation strategies delineates a specific emotional phenotype: men who are unable to tolerate distress internally and instead attempt to re-establish emotional control through outward dominance or annihilation.

However, emotional regulation in perpetrators is not purely intrapsychic but embedded within cultural scripts of masculinity that frame violence as a rational or even inevitable reaction to emotional pain. Qualitative research demonstrates that perpetrators often construct justification narratives to reinterpret their violence as legitimate responses to relational provocation or moral violation (Fahs et al., 2023; Di Marco and Evans, 2021). These narratives typically invoke hegemonic masculinity (expectations of male authority, possession, and emotional invulnerability) as frameworks for sense-making. Denial, minimization, and victim-blaming are recurrent cognitive strategies that protect the perpetrator’s self-concept, allowing emotional equilibrium to be restored through moral disengagement. In this light, violence becomes both a product and a performance of culturally conditioned emotional regulation. Indeed, masculine socialization encourages emotional suppression and discourages the expression of vulnerability, reinforcing the belief that intimate partners should serve as the central site of emotional containment and identity stabilisation. This gendered affective conditioning not only heightens the affective intensity of perceived loss but also narrows the repertoire of available self-regulation strategies, increasing the likelihood of collapse into externalized aggression.

Recent theoretical developments reinforce this model by identifying asymmetries in male–female psychobiological architecture that make men uniquely vulnerable to relational instability. To understand how cultural fictionality may operate as an affective training mechanism, we draw on the Tie-Up Theory of human mating (Lucchi Basili and Sacco, 2016, 2017, 2020), which provides a sexually dimorphic model of long-term couple formation particularly suited to the analysis of the emotional precursors of IPV.

The theory posits that each individual possesses two functionally distinct systems governing romantic attraction and bonding. The Active Area (AA) mainly operates at the conscious level and is sensitive to social conditioning. It responds to culturally salient partner characteristics: what we “think” we want or “should” want in a partner. The AA is sexually dimorphic: the Male Active Area (M-AA) is oriented toward the sexual dimension (physical attractiveness, sexual access), while the Female Active Area (F-AA) is oriented toward the psycho-emotional dimension (relational intimacy, emotional connection). The AA conducts *Filter Tests*, i.e., evaluations based on social desirability criteria such as wealth, status, physical appearance, personality, and social conformity.

The Receptive Area (RA) operates largely at the sub-conscious level and is not directly subject to social manipulation. It responds to compatibility signals that may contradict conscious preferences. The RA is sexually dimorphic in the opposite configuration: the Male Receptive Area (M-RA) is oriented toward the psycho-emotional dimension (personality compatibility, emotional resonance), whereas the Female Receptive Area (F-RA) is oriented toward the sexual dimension (biological compatibility, physical chemistry). The RA conducts the *Compatibility Test*, a sub-conscious assessment that, when successfully passed, paves the way (but not necessarily determines) what the theory terms a *Tie-Up (TU)*: a durable, involuntary emotional bond to a specific partner perceived as unique and irreplaceable.

If the partners also successfully navigate the *Tie-Up Cycle (TU-C)*, a specific sequence of reciprocal direct rewards (from AAs) and indirect rewards (from RAs), their interaction becomes self-catalytic, generating the super-cooperation characteristic of stable, resilient long-term couples, which, once sufficiently iterated, induces the Tie-Ups if they have not fully formed yet as a result of the successful Compatibility Tests. When both partners successfully engage with the TU-C, a *Double Tie-Up (D-TU)* emerges; note that the two partners may tie-up at different stages of the process.

The M-RA’s psycho-emotional orientation means that men tie-up based on assessments of personality compatibility and emotional connection with their female partner, not primarily on sexual attraction (which is the domain of the M-AA). This creates a paradoxical vulnerability: men are most emotionally dependent precisely in the dimension (psycho-emotional) where patriarchal conditioning provides them the least support and fewest alternative regulatory resources (Lucchi Basili and Sacco, 2025).

Empirical evidence shows that men rely disproportionately on romantic partners as their primary, and often exclusive, source of emotional intimacy, psychological support, and identity validation (Wahring et al., 2024). When the M-TU forms, possibly unilaterally, without adequate psycho-emotional skills or alternative support networks, the male partner becomes critically dependent on the relationship for emotional regulation. Threats to this bond such as perceived rejection, partner autonomy, or separation, generate overwhelming tension in the M-RA that the under-resourced male emotional system cannot process adaptively.

Moreover, when the M-AA and M-RA are in *opposition*, that is, when Filter Tests fail (the partner does not meet social standards of desirability or submissiveness) whereas the Compatibility Test succeeds and the M-TU has formed, an internal conflict emerges. The male subject experiences simultaneous emotional dependence (from the M-RA) and conscious disapproval (from the M-AA), a configuration associated with particularly volatile relationship dynamics in the Mating Stability Matrix framework (Lucchi Basili and Sacco, 2020).

This framework allows us to understand how cultural fiction might operate preventively. First, narratives may train the RA. Narratives that depict psycho-emotional complexity, male vulnerability, and relational negotiation engage the M-RA where men form their emotional bonds, potentially increasing coping with delusion in this domain. Second, they may facilitate the recalibration of Filter Tests. Fiction that presents alternative masculine models (where vulnerability is not punished, where distributed emotional support is valued) can reshape the M-AA’s socially conditioned criteria, reducing the AA/RA opposition. Third, they may expand the regulatory resources of men. Stories depicting male characters who maintain emotional wellbeing through friendships, creative pursuits, and non-romantic intimacy demonstrate that the M-RA need not rely exclusively on romantic partners for psycho-emotional rewards. And finally, they may train men to better tolerate frustration. By simulating relational disappointments, rejections, and losses within safe aesthetic frames, fiction allows a rehearsal of adaptive responses to violated expectations, that is, the very situations that trigger escalation in under-regulated male subjects.

In the subsequent sections, we show how this framework integrates with Predictive Processing neuroscience to explain the mechanisms through which cultural participation may interrupt the pathway from emotional dysregulation to violence.

## 3 Methodology

This paper adopts a theoretical and integrative methodology aimed at developing a conceptual model linking cultural fictionality to emotional regulation processes associated with gendered violence and feminicide. Although no original empirical data are collected, the study follows established procedures for conceptual research in psychology and cultural theory.

A structured interdisciplinary review was conducted across psychology, cognitive neuroscience, media studies, feminist theory, and violence studies. Searches were performed in Scopus and Web of Science or via Elicit, prioritizing peer-reviewed studies, in order to identify empirical and theoretical contributions relevant to four interconnected domains: (1) emotional and cognitive precursors of feminicide and intimate partner violence; (2) Predictive Processing and the Free Energy Principle as models of affective regulation, interoception, and prediction-error minimization; (3) fiction, narrative engagement, empathy, identification, and the effects of cultural participation on emotional regulation; and (4) feminist, intersectional, and masculinity studies relevant to gendered affective conditioning. Search terms combined keywords from the above-mentioned dimensions. The selected literature was then organized through a thematic synthesis, grouping findings into four analytical clusters: (a) multifactorial determinants and emotional escalation in feminicide; (b) predictive and interoceptive models of emotional dysregulation; (c) empathy, identification, and narrative simulation in fiction; and (d) gendered affective conditioning and male emotional dependence. Findings were integrated to construct a predictive-affective model that interprets cultural fictionality as a form of emotional training. This integrative step forms the basis for the paper’s theoretical contribution. The final phase translated the conceptual model into preventive implications for cultural policy and educational programs.

Our analytical approach to cultural fictionality builds on the narrative simulation methodology developed in Tie-Up Theory research (Lucchi Basili and Sacco, 2017, 2018, 2019). This methodology treats socially validated fictional narratives as ‘experiments’ that test how specific character configurations, defined by the concordance/opposition of their Active and Receptive Areas, and by the success/failure of their Compatibility and Filter Tests, navigate mating-related challenges.

Unlike experimental psychology, which necessarily abstracts partner characteristics into categorical variables, narrative analysis preserves the processual, context-dependent nature of actual mating interactions. A character who ‘successfully passes the Compatibility Test’ in a narrative context demonstrates not the presence of abstract desirable traits, but the capacity to engage the opposite-sex character’s RA through authentic interaction within a specific relational history.

This complementarity is crucial for understanding gender violence prevention. Experimental studies identify *population-level risk factors* (e.g., prior IPV, separation threats, resource stress), while narrative analysis illuminates *individual-level pathways*, such as how a specific male subject’s particular configuration of M-AA/M-RA concordance, Filter Test outcomes, and TU-C dynamics leads to violent versus non-violent outcomes under similar external pressures.

For prevention through cultural participation, this distinction matters: we are not training men to avoid categorical risk factors, but to develop the emotional flexibility to navigate the inevitable frustrations of actual, specific romantic relationships without defaulting to control-restoration behaviors.

As this manuscript was conceived as a Hypothesis and Theory article, the purpose of the review was not an exhaustive coverage or meta-analytic assessment, but the identification of robust cross-disciplinary convergences that may support a novel conceptual framework. Nevertheless, the revised description of search domains, inclusion logic, and thematic clustering is intended to improve transparency, evaluability, and reproducibility. As a conceptual synthesis, the model remains exploratory and requires empirical validation. Future research in experimental psychology, audience studies, and intervention-based work is needed to test whether fictional engagement modulates predictive dynamics and emotional regulation in populations at risk of gendered aggression.

## 4 Theoretical background

### 4.1 Predictive brain

All biological organisms face a fundamental challenge: maintaining their internal organization against the universal tendency toward disorder (entropy). The *Free Energy Principle* (FEP), formulated by Friston (2010), provides a formal mathematical framework for understanding this imperative. According to the FEP, organisms can be understood as systems that minimize “free energy,” a quantity measuring the divergence between an organism’s internal model of the world (its predictions or beliefs) and the sensory evidence it actually receives.

High free energy indicates that current sensory inputs are unexpected or incompatible with the organism’s predictions, signaling potential threats to the system’s integrity. By continuously working to minimize free energy, organisms effectively reduce uncertainty about the causes of their sensory inputs, a process termed *self-evidencing* (Hohwy, 2013).

The human brain instantiates this principle through a hierarchical architecture of prediction and error correction. Rather than passively receiving and processing sensory information, the brain continuously generates predictions about incoming sensory data at multiple levels of abstraction, from basic perceptual features to complex social scenarios. These top-down predictions are compared against actual bottom-up sensory inputs, and any mismatch generates *prediction error*, a signal propagated up the hierarchy to update higher-level models. This framework is known as *Predictive Processing* (PP), with *Active Inference* (AI) as its extension to action and decision-making (Clark, 2013; Friston et al., 2017; Hohwy, 2013).

The brain minimizes prediction error through three complementary mechanisms. First, *perceptual inference*: updating internal models to better align with sensory evidence. Second, *active inference*: modifying the world through action to generate sensory inputs that confirm existing predictions. Third, *precision weighting*: modulating the relative influence of predictions versus prediction errors by estimating the reliability (precision) of each. When sensory signals are noisy or unreliable, the brain assigns higher precision to its predictions, effectively trusting its model over ambiguous data. Conversely, when sensory signals are clear and informative, prediction errors receive higher precision, driving rapid model updating. This mechanism, mediated neurobiologically by attention and neuromodulation, determines the balance between stability and flexibility in cognitive processing (Feldman Barrett and Simmons, 2015; Clark, 2013).

This architecture explains a wide range of phenomena, from basic perception to complex cognition, but its implications extend profoundly into the emotional and social domains.

### 4.2 Prediction error and emotion

The Predictive Processing framework offers a powerful lens for understanding affective experience and emotional regulation. Within this model, emotions arise from the brain’s continuous attempt to predict and regulate the body’s internal physiological states (interoception) in relation to environmental demands and opportunities (Seth and Friston, 2016). Specifically, emotions emerge when there is a salient mismatch between *predicted* interoceptive states (how the body should feel given the current situation and goals) and *actual* interoceptive signals (how the body currently feels).

Consider an unexpected romantic rejection. The brain had predicted a certain pattern of interoceptive signals associated with continued intimacy and social bonding (calm cardiovascular state, certain patterns of autonomic arousal). The rejection generates a cascade of prediction errors: actual interoceptive signals (increased heart rate, stress hormone release, disrupted reward signaling) violate these predictions. The subjective experience of this sustained prediction error, i.e., the feeling that something is fundamentally wrong, that the world is not as it should be, is what we recognize as emotional distress.

From this perspective, emotional regulation corresponds to the capacity to tolerate, modulate, and adaptively respond to interoceptive prediction errors without rigid suppression or chaotic amplification (Ridderinkhof, 2017). Effective emotion regulation involves flexibly adjusting precision weighting: sometimes updating our predictions about how we should feel (acceptance, reappraisal), sometimes taking action to change our circumstances and thereby restore expected interoceptive states (problem-solving, seeking support), and sometimes simply maintaining our model while tolerating temporary prediction error (distress tolerance).

Ridderinkhof (2017) demonstrates that emotions function as states of *action readiness*: organized patterns of physiological and cognitive preparation that orient the organism toward prediction error minimization. When an event is appraised as threatening one’s goals or wellbeing (generating large prediction errors), the emotional system prepares specific action tendencies: fear prepares flight or freeze, anger prepares confrontation or boundary defense, sadness signals loss and may prepare withdrawal or help-seeking. These are not arbitrary reactions but evolved solutions to recurrent adaptive problems, implemented through the predictive architecture.

Crucially, this process involves *forward modeling*: the brain simulates the interoceptive and environmental consequences of different potential actions *before* executing them. A man whose partner announces separation can simulate (predict) the interoceptive outcomes of various responses: accepting the decision and grieving (predicting intense but manageable distress), pleading for reconsideration (predicting hope mixed with uncertainty), or attempting coercive control (predicting temporary reduction in the intolerable uncertainty, though this prediction may be based on maladaptive models). Emotional regulation, in this framework, is the process of using these forward models to select actions that will minimize long-term prediction error while avoiding short-term maladaptive error-reduction strategies.

The predictive framework thus reframes emotional dysregulation not as a failure of control, but as a failure of adaptive model updating and flexible precision weighting. When individuals assign excessive precision to their predictions about how relationships should unfold (high confidence in expectations of permanence, control, or reciprocation), actual evidence of partner autonomy, preference change, or departure generates catastrophic prediction error. If the individual’s predictive model is too rigid to update (unable to revise beliefs about relationship permanence or entitlement), and if active inference is constrained by social reality (the partner cannot be forced to stay through action), the only remaining option within a maladaptive framework becomes eliminating the source of intolerable error, either by eliminating awareness of it (denial, dissociation) or, in the most tragic cases, by eliminating the other person.

### 4.3 Predictive rigidity and masculinity scripts

The mechanism of *precision weighting*, that is, the brain’s estimation of confidence in its predictions versus sensory signals, is central to understanding both adaptive and pathological emotional regulation (Feldman Barrett and Simmons, 2015; Friston, 2010). This process determines the balance between model stability (trusting accumulated experience) and model flexibility (updating based on new evidence).

When the brain assigns excessively high precision to its internal predictions, it becomes *prediction-rigid*: sensory evidence that contradicts the model is discounted as noise, and the model persists unchanged despite accumulating errors. In perception, this can produce hallucinations (seeing things that aren’t there because internal predictions override sensory evidence). In social cognition, this produces dogmatism, confirmation bias, and the inability to update beliefs about relationships despite clear disconfirming evidence.

Conversely, when the brain assigns excessive precision to sensory inputs while under-weighting its predictions, experience becomes *prediction-unstable*: every fluctuation in sensation drives model revision, producing anxiety, hypervigilance, and the inability to maintain coherent expectations. The system oscillates chaotically, unable to settle on stable beliefs about self, others, or the future.

Adaptive emotional regulation requires dynamic precision weighting in terms of the capacity to flexibly modulate confidence in predictions versus evidence according to context (Clark, 2013). In safe, predictable situations, high precision on predictions enables efficient processing and stable expectations. In novel or rapidly changing situations, increased precision on prediction errors enables learning and adaptation. Critically, this balance must be continuously recalibrated based on a meta-cognitive assessment of reliability.

In the context of IPV, maladaptive precision weighting manifests in two complementary forms. The first is predictive rigidity regarding relational entitlement: when patriarchal socialization leads men to assign extremely high precision to predictions about male authority, female compliance, and relationship permanence, these predictions become resistant to updating. A partner’s assertion of autonomy, her expression of dissatisfaction, or her decision to leave generates prediction error, but instead of prompting model revision (“perhaps my expectations were unrealistic”), excessive precision on the prior belief leads to alternative explanations: “she’s being irrational,” “she’s been manipulated by feminists,” “she’ll come back once she realizes her mistake.” The prediction error is attributed to temporary noise rather than fundamental model failure. The neurobiological implementation of this rigidity likely involves learned patterns of precision modulation in circuits connecting prefrontal cortex (which generates high-level predictions about social relationships) with subcortical structures (which signal prediction errors). Repeated cultural reinforcement of male dominance scripts, combined with punishment of masculine vulnerability, may train these circuits to maintain high precision on dominance-related predictions even in the face of contradictory evidence.

The second form is precision amplification of threat signals: simultaneously, men socialized into rigid masculine identities often assign excessive precision to any sensory signals that might indicate loss of control, status diminution, or relationship threat (Ridderinkhof, 2017). A partner’s neutral tone is interpreted as coldness, her time with friends as betrayal, her professional success as emasculation. This hypervigilance to perceived threats reflects not emotional sensitivity but maladaptive precision weighting: the brain treats ambiguous signals as highly informative evidence of feared outcomes.

This dual dysfunction of rigid predictions of entitlement combined with amplified detection of threats to those predictions creates a volatile combination. The man expects constant validation and control (high-precision prediction), interprets minor autonomy as major threat (high-precision error amplification), yet cannot revise his model of what he’s entitled to (prediction rigidity). Each cycle of perceived threat followed by control-restoration behavior that temporarily “works” (the partner complies due to fear) reinforces both the prediction and the precision weighting, making the pattern increasingly entrenched.

In terms of forward modeling and the prediction of violence, the predictive system’s capacity to simulate action outcomes before execution provides a potential intervention point but also a mechanism for escalation. A man in the escalation phase (see section 5.1 below) generates forward models of different responses to perceived rejection: seeking emotional support from friends (prediction: vulnerability and potential shame, which are highly aversive given masculine norms), accepting the loss and grieving (prediction: unbearable sustained distress), confronting the partner (prediction: temporary relief through reassertion of control).

If prior learning has established that controlling behaviors “reduce uncertainty” (even if ultimately maladaptive), the forward model predicts that escalated control under the form of surveillance, coercion, threats, violence, will minimize the intolerable prediction error of losing the relationship. Crucially, because these forward models operate over short time horizons and with poor models of others’ agency, they may genuinely predict that violence will “solve” the problem (she’ll stay, she’ll comply) while failing to accurately simulate longer-term consequences (permanent relationship destruction, criminal consequences, moral injury).

The implications for the predictive-affective training hypothesis are substantial. This analysis suggests that effective cultural intervention must target multiple levels of the predictive hierarchy:

1. *Reducing prediction rigidity*: Narratives that depict male characters revising their relationship expectations in response to evidence, modeling the cognitive process of updating rather than defending maladaptive beliefs.
2. *Recalibrating precision weighting*: Stories that show male characters learning to tolerate ambiguity and interpret ambiguous partner behavior with appropriate uncertainty rather than catastrophizing.
3. *Enriching forward models*: Fiction that simulates the actual long-term consequences of control versus acceptance, violence versus vulnerability, providing predictive learning that short-term experience fails to supply.
4. *Training emotional tolerance*: Repeated aesthetic exposure to prediction errors in the psycho-emotional domain (simulated rejection, loss, uncertainty) within safe narrative frames, allowing the predictive system to learn that such errors are survivable and need not trigger catastrophic responses.

In the following section, we examine how cultural fiction specifically provides this multilevel training through its unique properties as an affective simulator.

### 4.4 The tie-up paradox: when emotional connection becomes weaponized

A particularly troubling dimension of intimate partner feminicide emerges when we consider the role of the male Tie-Up (M-TU) itself. Tie-Up Theory posits that the M-TU, the male bond formed through successful psycho-emotional compatibility, may, within a context of Double Tie-Up, drive super-cooperation and mutual investment in the couple relationship. However, empirical evidence suggests that perpetrators often describe intense emotional attachment to their victims prior to lethal violence (Di Marco and Sandberg, 2024; Fahs et al., 2023).

This apparent contradiction reveals a critical distinction between *functional* and *pathological* M-TUs. In a functional M-TU operating within a successful Tie-Up Cycle (TU-C), the male subject’s psycho-emotional dependence on his partner is balanced by various factors. First, a bidirectional reward flow: within a self-reinforcing Tie-Up Cycle, men are not only receiving rewards but reward their partner in turn. Second, the management of frustration by adaptive precision weighting: the male subject can tolerate temporary disruptions in reward flow without catastrophic anxiety, as his predictive system assigns appropriate uncertainty to relational states. Third, the altruistic orientation fostered by the iterations of the TU-C, which generates genuine other-centeredness, where the partner’s wellbeing becomes intrinsically valuable rather than instrumentally necessary for self-regulation.

In contrast, a pathological M-TU emerges when the M-TU becomes the *exclusive* source of emotional regulation for the male subject, reflecting the extreme emotional dependency of male relational architecture. The pathological trajectory is predicted by specific configurations within the Tie-Up framework: (a) a unilateral M-TU that has formed without a reciprocal F-TU, depriving the relationship of the self-catalytic reward dynamics that sustain functional couples; (b) strong AA/RA opposition, where the M-AA’s socially conditioned criteria actively conflict with the M-RA’s compatibility assessment, generating chronic internal tension; and (c) an impoverished TU-C, where insufficient iterations of reciprocal reward have failed to develop the altruistic orientation that would reframe the partner’s autonomy as intrinsically valuable rather than threatening. This may be further compounded when the M-AA remains discordant with the M-RA, failing the key Filter Tests (e.g., the partner does not conform to social standards of desirability or submissiveness), creating internal conflict, and if patriarchal conditioning has eliminated alternative sources of psycho-emotional support (male friendships, family bonds, therapeutic relationships).

Under these conditions, the M-TU paradoxically *increases* the risk of violence. The male subject experiences his partner as simultaneously indispensable (due to extreme M-RA dependence) and inadequate (due to M-AA disapproval). Separation or perceived abandonment activates overwhelming prediction error that cannot be regulated through internal reappraisal or external support-seeking. Violence becomes a desperate attempt to eliminate the source of unbearable uncertainty, not because the perpetrator lacks an emotional bond to the victim, but because such bond has become his sole regulatory mechanism.

This framework explains the otherwise puzzling observation that many feminicides occur precisely when the male subject is most emotionally dependent, often following the partner’s announcement of separation. The M-TU, rather than protecting the relationship, has become a vulnerability that patriarchal conditioning and emotional isolation transform into a lethal liability. In this context, **f**ictions that depict male characters developing *distributed* sources of psycho-emotional regulation, including meaningful male friendships, mentorship relationships, and reflective solitude, may be particularly protective, reducing the catastrophic stakes of any single relational disruption.

## 5 Discussion: culture as predictive-affective simulator

The preceding sections have established two complementary accounts of vulnerability: Predictive Processing explains *how* emotional dysregulation arises (through rigid precision weighting, catastrophic prediction error, and impoverished forward models), whereas Tie-Up Theory specifies *where* men are most exposed (the psycho-emotional dimension of the M-RA, precisely the domain in which patriarchal conditioning provides fewest alternative regulatory resources). Their convergence yields a specific prediction: any intervention that would generate controlled prediction errors in the psycho-emotional domain (i.e., errors that are intense enough to engage the M-RA yet contained enough to permit adaptive updating rather than defensive rigidity) would address the mechanism at the point of greatest leverage. Cultural fictionality possesses exactly this dual property. Because fictional narratives activate affective and predictive processes that are functionally equivalent to those operative in real social situations (Oatley, 2016; Mar and Oatley, 2008), and yet do so within the symbolic safety of the aesthetic frame where real-world consequences are suspended, they constitute what we term a *predictive-affective simulator*: a structured environment in which the brain can rehearse precisely those interoceptive prediction errors (rejection, loss, ambiguity, frustrated expectation) that, when encountered without preparation in actual relationships, trigger the escalation pathway described above. In what follows, we examine the empirical and theoretical evidence for this claim, specifying the cognitive, neurobiological, and social mechanisms through which fiction exercises this regulatory function.

A critical clarification is necessary regarding the temporal logic of the model. The intervention we propose is prophylactic, not reactive: cultural fiction does not intervene during an active escalation crisis, but builds regulatory capacity over time, such that when relational disruption occurs, the individual possesses richer predictive models, more flexible precision weighting, and a broader repertoire of forward-modeled responses. The “intervention window” identified below (Section 5.1) designates the phase of the escalation sequence where the absence of such prior training becomes most consequential, i.e., where the gap between the regulatory demands of the situation and the individual’s available resources determines whether the trajectory bends toward adaptive coping or collapses into violence. Fiction’s contribution is to have narrowed that gap before the crisis arrives.

### 5.1 Interrupting the path to violence: the preventive intervention window

As we have seen from literature (Aguilar-Ruiz, 2018; Cáceres, 2022; Fahs et al., 2023), intimate partner femicide converges toward a critical insight: lethal violence against women is rarely an impulsive eruption of uncontrollable anger. Rather, it emerges through a progressive escalation that, reinterpreted through the Predictive Processing and Tie-Up Theory frameworks, can be articulated as four interconnected phases. The *trigger phase* begins when the perpetrator perceives a relational threat: rejection, abandonment, betrayal, loss of attention, or the partner’s assertion of autonomy. In Tie-Up Theory terms, these events generate acute prediction error in the M-RA, where the male subject’s deepest emotional bond is located; the initial disturbance is vulnerability, not rage, but patriarchal scripts discourage men from acknowledging or processing it. The *escalation phase* emerges when the individual lacks the regulatory resources to metabolize these destabilizing emotions. The predictive system, exhibiting the dual dysfunction described in Section 4.3 (rigid predictions of entitlement combined with amplified threat detection) fails to update its relational model. In this phase, fear and shame harden into anger, rumination, and cognitive narrowing.

The literature further shows that following relationship dissolution, men experience steeper declines in psychological wellbeing, greater loneliness, and a higher likelihood of maladaptive coping strategies such as substance use, withdrawal, and rumination (Morris et al., 2023). These dynamics mirror the “trigger” and “escalation” phases of the emotional dysregulation model. It is precisely between this phase and the next that a critical *intervention window* opens (Table 1), one that current prevention strategies rarely address: the potential of cultural fictionality to redirect the trajectory from maladaptive coping to internal emotional regulation.

**Table 1:** Preventive intervention window.

Phase	Description	Key emotional/cognitive mechanisms	Relation to feminicide risk
1. Trigger phase	Perceived relational threat (rejection, betrayal, loss, autonomy of partner).	Fear, shame, humiliation; violation of internal predictive models of control.	Creates emotional destabilization and vulnerability that patriarchal scripts do not allow to process.
2. Escalation phase	Failure to metabolize negative affect; intensification of internal conflict.	Rumination, anger, cognitive narrowing; predictive rigidity; intolerance of uncertainty.	Emotional overload increases the likelihood of externalized aggression as a means to restore control.
→ Preventive intervention window	Cultural fictionality as predictive-affective training (i.e., theatre, cinema, literature).	Exposure to disappointment, ambiguity, loss; safe emotional simulation; predictive updating; frustration tolerance; reflective delay.	Redirects escalation toward internal emotional regulation, reducing risk of collapse into violence.
3. Collapse phase	Breakdown of regulation; activation of maladaptive coping strategies.	Externalization, coercion, impulsivity; elimination of perceived threat.	Violence becomes a means to restore coherence and resolve intolerable prediction error.
4. Rationalization phase	*Post-hoc* narrative reconstruction of the act.	Justification scripts, hegemonic masculinity, victim-blaming; cognitive coherence restoration.	Reinforces cultural legitimacy of control-based violence and masks the underlying dysregulation.

The temporal logic of this window must be understood correctly: it designates the phase at which regulatory capacity (or its absence) becomes decisive, not a moment for real-time cultural intervention. A man whose predictive system has been trained, through sustained engagement with emotionally complex fiction, to tolerate psycho-emotional prediction error possesses richer forward models at this juncture: he can simulate outcomes beyond the short-term relief of control-restoration, including the long-term consequences of aggression and the survivability of loss. One whose system has not been so trained defaults to the impoverished forward models described in Section 4.3.

Without such resources, the individual may progress to the *collapse phase*, where maladaptive coping strategies such as substance use, impulsive acting-out, externalization, or violence, are employed in an attempt to restore a sense of coherence and control. Violence becomes a drastic means of resolving intolerable prediction error by eliminating the source of emotional disequilibrium. Finally, the *rationalization phase* involves the construction of *post-hoc* hegemonic narratives that socially justify the act, legitimize the perpetrator and restore cognitive order. Through this discursive work, the perpetrator integrates the violent act into a coherent, culturally intelligible sense of self.

The preventive efficacy of cultural fiction at this intervention window is rooted in the sexually dimorphic architecture of emotional regulation described by the Tie-Up Theory. During the escalation phase, the M-RA (Male Receptive Area) experiences mounting distress as the psycho-emotional connection, the primary source of indirect rewards for men, appears threatened. The M-AA (Male Active Area), oriented toward sexual rewards and highly sensitive to social conditioning around masculine dominance, interprets this threat through patriarchal scripts that frame control as the appropriate response.

Cultural fiction may interrupt this trajectory by providing simulated experiences that train the M-RA to tolerate prediction error in the psycho-emotional sphere without defaulting to dominance-restoration behaviors. Through repeated exposure to narratives where male characters experience rejection, loss, or partner autonomy *without* resorting to violence, and indeed, where such non-violent responses lead to meaningful indirect rewards, the predictive system can learn alternative pathways. Crucially, because the M-RA operates at the psycho-emotional level where men are uniquely vulnerable (Lucchi Basili and Sacco, 2020), fiction that engages this dimension has particular resonance for male emotional regulation.

In the proposed analytical framework, feminicide is understood not as a spontaneous loss of control but as the outcome of a progressive failure in emotional and predictive regulation, reinforced by gendered expectations that equate vulnerability with weakness and control with masculinity. The insertion of cultural fictionality between the escalation and collapse phases identifies a previously neglected site for preventive action: a moment where prior aesthetic engagement have cultivated emotional resilience and redirected dysfunctional regulatory patterns before they crystallize into violence. This analysis has direct implications for cultural policy: funding criteria, educational curricula, and media literacy programs should privilege fiction with demonstrated capacity to engage male emotional complexity over simplistic action-oriented narratives, particularly for adolescent and young adult male audiences during the critical periods of their relational script formation.

### 5.2 Aesthetic experiences as a safe cognitive-emotional laboratory

Having identified where in the escalation sequence the absence of regulatory capacity becomes decisive, we now examine the evidence for how cultural fiction builds that capacity. A growing body of research demonstrates that fiction engages psychological and neurocognitive mechanisms uniquely suited to emotional learning and regulation.

Recent evidence from cognitive neuroscience and psychology has advanced our understanding of how artistic and cultural experiences shape the mind, body, and social behavior. A growing body of research (Fancourt et al., 2026) demonstrates that engagement with the arts produces measurable effects on mental health and emotional regulation through interconnected psychological, neurobiological, and social mechanisms. Three major domains of mechanisms through which the arts affect human functioning have been identified: psychological and cognitive mechanisms, neurobiological and affective mechanisms and social and identity mechanisms.

On one hand, artistic engagement activates attentional focus, mental imagery, and cognitive reappraisal, allowing individuals to simulate alternative realities and experiment with different emotional outcomes. This process parallels predictive coding in the brain: art presents structured yet uncertain sensory and symbolic input that demands continuous updating of internal models. From a neurobiological perspective, neuroimaging studies show that aesthetic experience recruits networks involved in reward processing, emotion, and interoceptive regulation. The dopaminergic system (associated with motivation and pleasure) and the default mode and salience networks co-activate during artistic immersion, supporting emotional exploration in a safe physiological range. This neural coordination enables the individual to feel intensely without being overwhelmed, thereby exercising tolerance to uncertainty and frustration. This is particularly evident in participatory forms of art that enhance synchronisation, empathy, and group belonging. The body itself becomes a regulatory interface, mediating emotional co-regulation and fostering collective resilience. The theory of embodied simulation developed by Gallese (2005, 2007) provides a crucial neuroscientific basis for understanding empathy and aesthetic engagement. Perceiving others’ actions or emotions activates neural patterns in the observer similar to those used when performing or feeling them, a process mediated by the mirror neuron system (Gallese, 2009). It should be noted that the scope and specificity of mirror neuron involvement in higher-order empathy remains debated (Hickok, 2014); however, the broader principle of embodied simulation that understanding others involves partially re-enacting their states is well supported across multiple methodologies. From a predictive perspective, such mirroring constitutes an anticipatory embodied simulation that enables a pre-reflective, bodily understanding of others and supports social regulation (Gallese, 2011). In narrative contexts, these mechanisms generate aesthetic empathy: during reading or film viewing, individuals vicariously experience characters’ emotions within a symbolic and safe space (Gallese and Guerra, 2015), making of fiction an affective training ground rather than mere entertainment. The arts may thus serve as shared affective regulators, reinforcing prosocial and empathic tendencies that counteract isolation and control-based dynamics often associated with gendered aggression.

Within this broader aesthetic domain, cultural fictionality constitute the most structured and immersive form of such simulation, as they uniquely combine perspective-taking, embodied emotional modeling, and narrative unpredictability, thereby providing an especially potent environment for practicing regulatory skills. Central among these mechanisms is experience-taking, defined as the imaginative process through which individuals temporarily relinquish access to their own self-concept in order to simulate a character’s thoughts, emotions, and goals (Kaufman and Libby, 2012). The suspension of disbelief is a predictive act: the brain negotiates between real and imagined worlds, practising flexibility, empathy, and tolerance of uncertainty. Neuroimaging evidence corroborates this mechanism: identifying with fictional characters produces measurable self–other neural overlap in the ventromedial prefrontal cortex (vMPFC), indicating shared neural substrates for processing one’s own emotional states and those of a character (Broom et al., 2021). Emotional engagement deepens this simulation process. Studies show that when individuals care about or feel close to characters, they simulate the characters’ subjective experiences, generating empathy, emotional contagion, and affective resonance (Teasdale et al., 2021; De Graaf et al., 2012). Importantly, audiences can maintain cognitive distance while emotionally engaged. This configuration mirrors therapeutic exposure: intense affect is activated but contained in a safe symbolic frame, that’s why we insist in talking about fictionality as a “safe space.” Identification with fictional characters can shift self-concepts, promote more favorable attitudes toward outgroups, reduce stereotyping, and influence real-world behaviors, including, for instance, voting choices several days after exposure (Kaufman and Libby, 2012). Such changes highlight that narrative immersion can recalibrate affective and cognitive dispositions, not merely while being exposed to the narrative but across time. Moderating factors identified across studies show how fiction can maximize emotional training effects. First-person narratives, internal perspectives, and delayed revelation of outgroup membership significantly enhance experience-taking and reduce stereotype application (De Graaf et al., 2012). Reduced self-concept accessibility facilitates deeper identification, while moral approval of characters strongly predicts emotional resonance (Teasdale et al., 2021).

In other words, during engagement with fiction, the predictive mind continuously formulates expectations about narrative developments, relationships, and emotional outcomes. When these expectations are violated, the resulting feelings of sadness, anger, confusion, or relief activate mechanisms of precision re-weighting and model updating: the brain learns to accommodate uncertainty rather than resist it. This process mirrors emotion regulation strategies described in acceptance-based therapies, where remaining with discomfort rather than avoiding it fosters resilience and cognitive flexibility. Within the symbolic safety of the aesthetic frame, cultural fiction thus may function as a collective neurocognitive training ground, allowing individuals to practice tolerating prediction error, emotional dissonance, and loss.

It should be noted that the evidence for “functional equivalence” between fiction-induced and real-world affective processing, while substantial, derives largely from laboratory studies using short text passages or film clips. The degree to which these findings generalize to sustained engagement with complex, long-form narratives such as novels, serialized drama, or theatrical performances remains an important empirical question, and one that future research should address using ecologically valid designs.

Through repeated exposure to simulated experiences of rejection, ambiguity, and moral conflict, the predictive system may refine its capacity for adaptive regulation, an essential capacity for preventing the escalation of frustration into aggression. Fictionality, in this sense, provides a safe cognitive-emotional laboratory where the mind can rehearse non-violent responses to violated expectations and learn that uncertainty and loss can be overcome.

This interpretation is reinforced by Oatley’s cognitive theory of fiction, which conceptualizes narratives as mental simulations of social life. Fiction allows readers to explore intentions, emotions, and relational dynamics, continuously updating predictive models of human interaction (Oatley, 2016). Through such simulated experiences, the predictive mind may gradually recalibrate the balance between internal expectations and affective feedback, developing tolerance for violated predictions. Within this framework, cultural fictionality provides a neurocognitive environment for retraining maladaptive mechanisms by routinely activating receptive emotional mechanisms. Engagement with fictional narratives in which frustration, abandonment, and moral conflict are symbolically rehearsed may facilitate the capacity to reappraise loss, delay reactive impulses, and transform emotion into reflection.

### 5.3 Ideological content vs. narrative structures that engage male emotional competence

As we have seen, violence emerges not only from personal dysregulation but from a broader ecosystem of stories, images, and symbolic frameworks that encode control, possession, and emotional entitlement as masculine norms (justification narratives). To have an integral view of such a complex phenomenon, it is fundamental to clarify whether ideological typology or the affective engagement and narrative immersion afforded by cultural fiction exert stronger influence on emotional regulation mechanisms that can either reinforce or mitigate the pathways leading to gendered aggression and feminicide.

Empirical research across psychology, media studies, and gender scholarship indicates that the ideological framing of cultural content has small-to-moderate but consistent effects on gender attitudes and empathy. Patriarchal or gender-stereotypical portrayals tend to reinforce traditional role beliefs and dominance-oriented scripts: meta-analytic evidence shows modest correlations between stereotyped television exposure and traditional gender norms (Hermann et al., 2022), while studies on music and gaming report heightened sexist attitudes among adolescents exposed to sexualized lyrics (Ter Bogt et al., 2010) and reduced empathy in men playing violent or sexist games (Gabbiadini et al., 2016). Conversely, feminist or counter-stereotypical narratives are associated with more egalitarian orientations. Reading erotica featuring dominant female protagonists increases women’s self-efficacy and reduces gender double standards (Harris et al., 2017), and participatory theatre programs addressing domestic violence have yielded measurable reductions in spousal abuse (Hoff et al., 2021). Literary fiction also shows modest associations with egalitarian gender attitudes and perspective-taking (Fong et al., 2015). However, these effects are moderated by identification, pre-existing beliefs, and narrative engagement: for example, the negative impact of sexist video game content is substantially reduced when controlling for baseline attitudes (Ferguson and Donnellan, 2017). Overall, ideological content shapes gender-relevant attitudes and empathy, but its influence remains contingent and context-dependent. Studies consistently show stronger effects among male audiences, where identification with dominant or aggressive characters reinforces gender hierarchies (Gabbiadini et al., 2016). Identification and interpretation are key moderators: patriarchal messages framed as “romantic” can normalize coercion, while critical or feminist framing fosters detachment and reflective distancing. Educational level and media literacy mediate outcomes: higher critical awareness correlates with weaker ideological effects (Behm-Morawitz and Mastro, 2008). Exposure dose and repetition further amplify attitudinal change, as prolonged contact consolidates internalised schemas (Hermann et al., 2022). Although no study directly links cultural consumption to feminicide, empirical evidence identifies cognitive-affective pathways connecting media exposure with attitudes that align with known risk correlates of IPV (Campbell et al., 2017; Ruíz, 2019).

From the perspective of the Tie-Up Theory, not all cultural fiction is equally effective in stimulating the male Receptive Area in ways that promote adaptive emotional regulation. The distinction between ideological content and narrative structure can be clarified in terms of their respective targets: ideological framing primarily operates on the M-AA (the conscious, socially conditioned system), shaping attitudes about gender roles and relational norms, whereas narrative immersion and affective engagement operate on the M-RA (the mostly sub-conscious system of psycho-emotional compatibility), training the very regulatory mechanisms where men are most vulnerable. This is why narrative structure may produce deeper and more durable effects: it reaches the level of processing where emotional bonds form and where regulatory failure originates. The framework suggests the necessity to attune the M-RA to psycho-emotional complexity, depth, and authenticity, that is, qualities often gendered as ‘feminine’ in patriarchal cultures and consequently devalued in male-oriented entertainment.

In particular, narratives most likely to train male frustration tolerance and prediction error management share specific structural features. First, giving salience to interiority and not only action: **s**tories that privilege character psychology over plot mechanics train the M-RA to respond to psycho-emotional nuance. Male characters who articulate inner conflict, vulnerability, and uncertainty model the kind of reflective engagement that enables regulation. Second, giving salience to the consequentiality of relational engagement. Narratives where male characters’ emotional choices have genuine psycho-emotional (not just plot) consequences for themselves and others activate the forward-modeling capacity that is essential for adaptive regulation. Third, valuing intimacy in male-to-male relationships: fiction portraying male friendships, father-son relationships, and mentor bonds as sources of genuine psycho-emotional reward and not merely instrumental alliances train the M-RA to count on distributed regulatory affective resources. And finally, unsanctioned vulnerability, where male characters who express fear, sadness, or uncertainty need not be retributed with narrative punishment (emasculation, defeat, ridicule). This kind of narrative input provides schema-inconsistent exemplars that challenge patriarchal conditioning.

Conversely, narratives that reinforce dominance-restoration scripts typically feature: male protagonists whose emotional regulation occurs through conquest or control; female characters who exist primarily to validate male identity; conflict resolution through domination rather than negotiation; and punishment of male vulnerability in terms of plot consequences.

In this perspective, findings indicate that contemporary cultural production is bifurcated into two antagonistic narrative regimes: stories that punish male vulnerability, thereby reinforcing patriarchal scripts of dominance and emotional suppression, and stories that validate vulnerability as a legitimate affective state, fostering reflective engagement and adaptive emotional regulation.

In terms of impact, while ideological content has small-to-moderate effects on attitudes, narrative structures that engage male emotional competence may produce deeper and more durable changes, fostering empathy, perspective-taking, and affect regulation (Oatley, 2016). However, no study has directly compared the two within a single experimental design, and the relative magnitude of their effects remains an empirical question. What the existing evidence does suggest is that ideological content primarily shapes conscious attitudes (operating through the M-AA), whereas narrative immersion may additionally recalibrate the subconscious regulatory processes (operating through the M-RA) that are most directly implicated in the escalation pathway. Moreover, even modest ideological effects become substantial when amplified through repetition, social diffusion, and intergenerational transmission across populations.

Integrating the predictive-affective framework with recent findings on male emotional escalation preceding feminicide (Di Marco and Sandberg, 2024; Amaoui et al., 2023) highlights how aesthetic engagement can serve a preventive and re-educational function. This process strengthens regulatory flexibility and empathy, core capacities that counteract the cognitive-affective rigidity underlying gendered violence.

## 6 Conclusion, implications, and limitations

The arguments developed throughout this article converge on a central hypothesis: the preventive potential of culture against gendered violence lies primarily in its ability to retrain emotional and predictive mechanisms. Ethically and emotionally complex aesthetic experiences, which amplify the contradiction rather than resolve it, train the predictive mind to endure dissonance and ambiguity. In feminist terms, empathic resonance erodes the illusion of separateness and domination, while emotional regulation teaches tolerance of vulnerability, a capacity systematically suppressed by patriarchal systems.

At the same time, the framework’s level of claim must be clearly delimited. Although it is oriented toward prevention, at this stage the model does not argue that engagement with fiction directly prevents feminicide; rather, it is a hypothesis-generating proposal targeting intermediate variables empirically associated with IPV risk. The systematic operationalization of these constructs into validated indicators, experimental paradigms, and intervention protocols remains an essential next step and will require dedicated *ad hoc* empirical research. For instance, predictive rigidity may be assessed via established measures of intolerance of uncertainty and cognitive inflexibility; distress tolerance through validated self-report instruments and behavioral frustration paradigms; hostile masculinity through standardized attitudinal scales; and narrative training effects through pre-post experimental designs examining changes in empathy, emotion regulation strategies, and responses to simulated rejection or relational threat. Neurocognitive correlates could also be explored using emotion-regulation tasks indexing prefrontal control and interoceptive processing. In this sense, the present paper functions as a conceptual scaffold for future empirical testing and translational development, rather than as evidence of direct preventive efficacy.

Moving from conceptual work to implementation, however, requires enabling cultural and institutional conditions. Before translating insights and results into practice, a feminist cultural policy ecosystem is required, that recognizes the arts as resources for emotional literacy and social prevention. This means supporting creative environments that cultivate empathy, ambiguity, and interdependence rather than competition or closure. Arts education should be reoriented toward empathetic learning, using fictional creative activities that make people “stick with their emotions”, and then dwell on them toward a path of self-introspection. Cultural institutions, museums, schools, and media platforms can become laboratories of emotional democracy, where citizens learn to inhabit contradiction and difference without annihilation. Preventive programs addressing male violence could integrate aesthetic and narrative interventions that help participants process emotions of shame, loss, and rejection through symbolic exploration and reenactment rather than control. Such programs, grounded in the predictive-affective model, would complement cognitive-behavioral and psychodynamic approaches by emphasizing embodied regulation and empathic attunement.

Ultimately, a feminist cultural policy inspired by predictive processing envisions the cultural sphere as emotional infrastructure: a collective system for rehearsing coexistence, care, and mutual recognition. In this view, the arts become core technologies of prevention, cultivating the psychological flexibility and empathic competence necessary for a non-violent, interdependent world. Cultural fictionality thus emerges as a feminist technology of care, supporting emotional resilience and reducing control-based scripts that sustain domination.

This conclusion also reinforces a broader preventive premise: interventions must operate systemically, combining micro-level emotional regulation with macro-level attention to structural inequalities and gendered socialization. Violence indeed is an affective technology of domination that emerges when predictive, emotional, and empathic regulation fails. Accordingly, cultural participation can be framed as an ethical training ground that complements, rather than replaces, legal, educational, and psychosocial strategies. From a fourth-wave feminist standpoint (Kakroda and Sole, 2023), defined by transnational, intergenerational, and intersectional “hashtag feminism,” call-out practices, and the reconfiguration of sisterhood as an anti-essentialist alliance built through digital/embodied networks, this capacity has deep political significance: it challenges the patriarchal demand for control and closure, the psychic infrastructure that underlies domination, possessiveness, and gendered violence.

Notably, in the 4th-wave the female body returns as the key site of struggle where white (Collins, 2022), heterosexual citizenship and the modern patriarchal norm are (re)inscribed; the backlash on abortion and gender-based violence shows that governance of women’s bodies remains central to state formation under neoliberal and neo-fundamentalist pressures (Peroni and Rodak, 2020). The new wave redefines activism through transnational, intergenerational, and intersectional networks that merge digital and embodied practices, what Munro (2013) describes as “#feminism” and what Peroni and Rodak (2020) interpret as the reconfiguration of sisterhood into an anti-essentialist alliance based on empathy, autonomy, and shared vulnerability via hybrid spaces.

The Tie-Up Theory framework contributes a final, important insight to this political vision. The M-RA’s psycho-emotional orientation means that men’s capacity for deep emotional bonding is not a deficit to be managed but a resource to be cultivated, one that patriarchal systems have systematically impoverished by confining it to a single relational channel. A feminist cultural policy that enriches male psycho-emotional life through distributed sources of connection and meaning does not merely reduce violence risk; it expands the conditions for genuine intimacy, reciprocity, and cooperative partnership. Prevention and flourishing, in this framework, are not opposed objectives but two aspects of the same developmental trajectory.

## Data Availability

Publicly available datasets were analyzed in this study. This data can be found at: literature chapter and references.
